# The association between serum complement 4 and relapse of primary membranous nephropathy: a multicenter retrospective cohort study

**DOI:** 10.3389/fmed.2024.1451677

**Published:** 2024-11-11

**Authors:** Wenyuan Gan, Fan Zhu, Xingruo Zeng, Wei Xiao, Xun Fang, Licong Su, Wenli Chen

**Affiliations:** ^1^Department of Nephrology, The Central Hospital of Wuhan, Tongji Medical College, Huazhong University of Science and Technology, Wuhan, China; ^2^Division of Nephrology, Nanfang Hospital, Southern Medical University, National Clinical Research Center for Kidney Disease, State Key Laboratory of Organ Failure Research, Guangdong Provincial Institute of Nephrology, Guangdong Provincial Key Laboratory of Renal Failure Research, Guangzhou, China

**Keywords:** primary membranous nephropathy, relapse, serum complement 4, prediction model, multicentral study

## Abstract

**Background:**

Relapse after initial remission reduces renal survival in patients with primary membranous nephropathy (PMN). In this study, we aim to identify risk factors of relapse in PMN and construct a model to identify patients at high risk of relapse early.

**Methods:**

We conducted a multi-center retrospective study using the China Renal Data System database, which includes data from 24 urban academic centers across China. A prediction model based on the Cox proportional hazards model was derived in the derivation group and validated in the validation group.

**Result:**

515 patients with biopsy-proven PMN achieving initial remission were enrolled. 32.62% of patients subsequently relapsed during a median of 6.08 months. Lower serum albumin (Alb) (per 1 g/L decrease, hazard ratio [HR] =1.48, 95% confidence interval [CI] 1.29–1.78, *p* < 0.001), lower estimated glomerular filtration rate (eGFR) (per 10 mL/min/1.73m^2^ decrease, HR =1.14, 95% CI 0.97–1.49, *p* < 0.001), higher serum complement 4 (C4) (per 0.1 g/L increase, HR =1.89, 95% CI 1.32–3.22, *p* = 0.012), partial remission (PR) (HR =2.28, 95%CI 1.74–4.04, *p* < 0.001), and treatment with calcineurin inhibitors (CINs) (HR =1.33, 95%CI 1.04–1.64, *p* < 0.001) at the time of remission were risk factors for relapse. C-statistic, time-dependent areas under the receiver operating characteristic curve, and calibration plots confirmed that the model had excellent discrimination and calibration in predicting PMN relapse. The anti-phospholipase A2 receptor antibody (aPLA2Rab) titers and pathologic features did not substantially improve the model.

**Conclusion:**

Our study confirms the well-known low Alb and eGFR, PR, and treatment of CNIs at the time of remission as risk factors for PMN relapse, but aPLA2Rab and pathologic features may not predict relapse. In addition, it is the first study to show serum C4 is associated with PMN relapse. We suggest that complement-targeted therapies may be a potential therapy to prevent PMN relapse.

## Highlights

- There is little information on the relationship between clinical features after initial remission and relapse of primary membranous nephropathy (PMN) in the multicenter study.
- Our study is the first study to show serum C4 is associated with PMN relapse.
- We suggest that complement-targeted therapies may be a potential therapy to prevent PMN relapse.

## Introduction

Primary membranous nephropathy (PMN) is a leading cause of adult nephrotic syndrome and exhibits long natural course, its common pathological finding is the presence of subepithelial deposition over glomerular basement membrane (1). Its incidence in China has increased dramatically in recent years (2, 3). A total of 40–50% of patients achieve proteinuria remission while the remaining get deterioration of kidney function, step to the end stage of kidney disease (ESKD) in 5 to 10 years (4–6). Durable proteinuria remission reflects a clinical absence of disease activity and is associated with excellent long-term renal and patient survival, but relapse is associated with increased risk of loss of renal function (7, 8). Furthermore, patients who relapsed achieved remission again, which was lower than the rate observed in patients with initial remission (9). Therefore, it is essential to reduce the relapse rate of PMN after initial remission. However, there is little information on the relationship between clinical features after initial remission and relapse of PMN in multicenter studies in the Chinese region. Therefore, searching for risk factors to predict relapse, especially those based on clinical and pathological characteristics, is crucial for the treatment strategy of patients with PMN.

In this study, we retrospectively analyzed the clinical and pathological characteristics in patients with biopsy-proven PMN after the initial remission. Based on the analysis of a large cohort of patients in China Renal Data System (CRDS), we aimed to identify the risk factors of relapse in patients who achieved initial remission, and construct a model identifying patients with high relapse risk early and guiding management to decrease relapse risk.

## Methods

### Study design and data source

This is a multicenter retrospective cohort study using the de-identified data collected from the CRDS database from January 2000 to April 2023.^1^ The CRDS database is a joint initiative of the National Clinical Research Center for Kidney Disease and the China Center for Disease Control and Prevention, and formed by the regional medical centers across China. All of the laboratories of the participating centers had passed the annual External Quality Assessment by the Chinese National Center for Clinical Laboratories. The CRDS databases included data of more than 8 million patients from 24 large urban academic centres that cover the major geographic regions across China. The accuracy and completeness of this data base have been verified in our previous studies and by other validation activities (10–13).

### Study population

All patients with biopsy–proven PMN in the CRDS were eligible for study inclusion. The following inclusion criteria were applied: (1) Patients aged 18 years or over, with first presentation of PMN and subsequently achieve initial clinical remission; (2) Sufficient data on medical history, laboratory tests and treatment regimens. The exclusion criteria included: (1) Secondary membranous nephropathy caused by autoimmune diseases, hepatitis, malignancy, drugs and other systemic diseases; patients were excluded if the above diagnosis was recorded at any time throughout the course of the disease; (2) Kidney transplant patients; (3) Acute infection (acute respiratory/gastrointestinal infections) at the time of initial clinical remission. All diagnosis as classified according to the International Classification of Diseases, Tenth Revision, Clinical Modification (ICD-10-CM), the first 4 digits of ICD-10 to include all possible comorbidities.

### Study outcome

The primary outcome was relapse after initial remission. Complete remission (CR) defined as reduction of proteinuria to <0.3 g/d or urine protein creatinine ratio (PCR) <300 mg/g, stable renal function and serum albumin >35 g/L. Partial remission (PR) defined as reduction of proteinuria to 0.3–3.5 g/d or PCR 300–3,500 mg/g and a decrease >50% from baseline with stable renal function. A stable renal function was defined as estimated glomerular filtration rate (eGFR) <15 mL/min per 1.73 m^2^ decline from baseline. Relapse defined as proteinuria >3.5 g/d or PCR >3,500 mg/g after complete remission has been achieved or an increase in proteinuria by >50% during partial remission. The 24 h urine protein excretion was used first for analysis and PCR was used if the 24 h urine protein excretion was not available. For each patient, the date of initial clinical remission was considered as the index date. Follow-up was defined as the interval from initial remission to the onset of relapse or the last visit, whichever occurred first.

### Clinical variables

All variables were considered clinically relevant based on biological mechanism or evidence from previously published data. We collected demographic characteristics, comorbidities and pathological features at the time of renal biopsy. Pathological features included stages of PMN, glomerulosclerosis, crescents, tubular atrophy, interstitial fibrosis, and the glomeruli deposition of complement 3 (C3), C4 and immunoglobulin G (IgG). We extracted physical examination and laboratory data, including blood pressure, hemoglobin, serum albumin (Alb), serum creatinine (SCr), eGFR calculated according to the Chronic Kidney Disease Epidemiology Collaboration equation, 24 h urinary protein excretion, PCR, anti-phospholipase A2 receptor antibody (aPLA2Rab) titers as measured by enzyme-linked immunosorbent assay, serum C3, C4, and IgG at the time of initial clinical remission. The aPLA2Rab titter ≥20 RU/mL is considered as positive. We recorded therapeutic agents, including glucocorticoids, immunosuppressive drugs (azathioprine, cyclophosphamide, chlorambucil, cyclosporin, tacrolimus, mycophenolate mofetil, and rituximab), and other supportive medications (angiotensin–converting enzyme inhibitor [ACEi] and angiotensin receptor blocker [ARB]). Use of the above medications was defined as having at least one prescription of the drugs throughout the clinical course. We included the Anatomical Therapeutic Chemical codes with first 4 digits to capture all types of medications.

### Prediction model

We proposed a prediction model for predicting the relapse of PMN. To train and validate the performance of the prediction model, we divided the patients into the derivation and validation groups with a 7:3 ratio using random sampling method stratified by outcome events. Potential correlations and interactions between all variables in the prediction model were assessed to avoid collinearity problems. The benefits of the chosen variables were confirmed using a LASSO Regression. A prediction model based on the Cox proportional hazards model was established, and variables with *p* < 0.1 in the univariate analysis and biologically plausible variables were enrolled in the multivariate analysis. We use both forward and backward methods in our regression analyses, and show the results of the forward selection method if there is no difference between the final results of the two methods. The discriminative ability of the model was determined by C-statistic and the time-dependent areas under the receiver operating characteristic curve (AUROC). The calibration between derivation and validation cohorts was assessed using calibration plots.

### Subgroup and sensitivity analyses

Subgroup analyses stratified by age, sex, Alb, eGFR, remission type, and treatment regimens. An interaction term was added to test for possible effect modification by the grouping factors. Sensitivity analyses were performed to evaluate the robustness of the findings by restricting the endpoint defined by 24 h urinary protein excretion only, although this leads to a decrease in sample size.

### Statistical analysis

The t-test or Mann–Whitney U-test was used to compare normally or non-normally distributed continuous variables. The Chi-square test or Fisher’s exact test was used to compare categorical variables. Pearson correlation test or Spearman rank correlation test was used for correlation analysis. Cumulative incidence rates were calculated and plotted using Kaplan–Meier analysis, the comparison of incidence rates between groups were performed with a log-rank test. We managed missing data in all analyses using multiple imputation, with an assumption of missing at random. *p*-values were two-tailed and *p* < 0.05 was considered statistically significant. Statistical analysis was performed using R version 4.1.1 (R Foundation for Statistical Computing, Vienna, Austria) and employed packages survival, rms, pec, survminer, tidyverse, and dplyr.

### Ethics approval

The study protocol was approved by the Medical Ethics Committee of the Wuhan Central Hospital (approval number: 2021(3)-01), and Nanfang Hospital, Southern Medical University (approval number: NFEC-2019-213), and the requirement for informed consent was waived due to the retrospective nature of the study. This study was performed by the Strengthening the Reporting of Observational Studies in Epidemiology guidelines (14).

## Results

**Figure 1:**
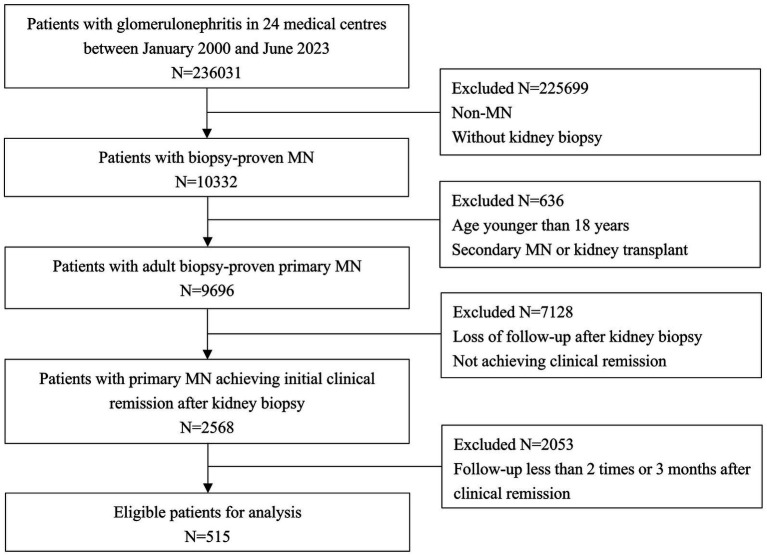
Flowchart of participant selection among 236,031 participants from 24 centers, 515 patients with biopsy-proven primary membranous nephropathy achieving initial remission were eligible for analysis.

Among 236,031 participants from 24 centers, 515 patients with biopsy-proven PMN achieving initial remission were eligible for analysis (Figure 1). The characteristics at the time of remission and follow-up were summarized in Table 1. All patients included in the study were followed for a total of 20.55 months (interquartile range [IQR] 17.52–22.93 months) from renal biopsy to the relapse or the last visit, of which the time from renal biopsy to remission was 7.26 months (IQR 5.13–8.27 months). The median follow-up time from remission to the relapse or the last visit was 13.68 months (IQR 7.21–20.15 months). 32.62% of patients (168/515) subsequently relapsed in a median of 6.08 months (IQR, 3.38–8.12 months). Compared with the non-relapse group, the relapse group was older (51.47 ± 12.37 vs. 46.63 ± 13.86 years, *p* < 0.001), had higher proteinuria (1.99 ± 1.45 vs. 0.16 ± 0.13 g/d, *p* < 0.001), lower Alb (17.22 ± 5.40 vs. 23.53 ± 5.89 g/L, *p* = 0.028), higher SCr (90.75 ± 39.91 vs. 73.69 ± 43.83 umol/L, *p* = 0.018), lower eGFR (85.60 ± 25.63 vs. 93.61 ± 25.90 mL/min/1.73m^2^, *p* = 0.001), and higher C4 (0.33 ± 0.09 vs. 0.27 ± 0.10 g/L, *p* = 0.032) at the time of remission. In addition, the occurrence of relapses was also significantly higher (72.02% vs. 27.98%) and earlier (6.07 vs13.43 months) in PR patients than in CR patients. A total of 432 (83.88%) patients were in stage I- II 432 (83.88%) and 83 (16.12%) patients were in stage III-IV. Crescent formation was found in 8.54%, focal and segmental glomerulosclerosis in 10.29% and tubular atrophy in 56.12%. IgG deposits were found in 94.95%, C3 deposits in 75.92% and C4 deposits in 18.06%. There were no difference in pathological characteristics between relapse group and non-relapse group (all *p* > 0.05). Of note, the renal biopsy data were from the onset of PMN, not from the time of remission, as there were no repeat renal biopsies data. A total of 278 patients were available for circulating aPLA2Rab titers, the mean titers was 25.25 RU/mL (IQR, 18.83–49.87 RU/mL), with no significant difference between the relapse and non-relapse groups (26.74 vs. 24.10 RU/mL, *p* = 0.082) at the time of remission, as show in Table 1.

**Table 1:** The characteristics at the time of remission and follow-up.

	All patients	Relapse group	Non-relapse group	*p* value
	(*n* = 515)	(*n* = 168)	(*n* = 347)	
Demographic characteristics				
Male no. (%)	354 (68.74%)	117 (69.64%)	237 (68.30%)	0.387
Age (years)	48.21 ± 13.57	51.47 ± 12.37	46.63 ± 13.86	<0.001*
Body mass index (kg/m^2^)	25.11 ± 3.11	25.38 ± 3.20	24.98 ± 3.06	0.178
Comorbidities				
Hypertension no. (%)	198 (38.45%)	67 (39.88%)	131 (37.75%)	0.310
Diabetes mellitus no. (%)	76 (14.76%)	30 (17.86%)	46 (13.26%)	0.212
Systolic blood pressure (mmHg)	131.12 ± 16.97	133.05 ± 18.32	130.19 ± 16.22	0.073
Diastolic blood pressure (mmHg)	80.95 ± 10.71	81.68 ± 11.18	80.60 ± 10.47	0.284
Laboratory tests				
Hemoglobin (g/L)	131.95 ± 21.61	132.36 ± 21.05	131.75 ± 21.91	0.765
Serum albumin (g/L)	22.43 ± 5.73	17.22 ± 5.40	23.53 ± 5.89	0.028*
Serum creatinine (μmol/L)	85.99 ± 42.68	90.75 ± 39.91	73.69 ± 43.83	0.018*
eGFR (mL/min/1.73 m^2^)	91.00 ± 26.06	85.60 ± 25.63	93.61 ± 25.90	0.001*
Triglyceride (mmol/L)	3.34 ± 8.82	3.11 ± 2.12	3.45 ± 10.65	0.566
Total cholesterol (mmol/L)	8.58 ± 2.97	8.65 ± 2.80	8.60 ± 2.91	0.777
Serum complement 3 (g/L)	1.13 ± 0.31	1.12 ± 0.42	1.13 ± 0.35	0.935
Serum complement 4 (g/L)	0.29 ± 0.11	0.33 ± 0.09	0.27 ± 0.10	0.032*
aPLA2Rab titer (RU/mL) ^#^	25.25 (18.83, 49.87)	26.74 (17.02, 52.2)	24.10 (16.58, 48.52)	0.082
aPLA2Rab positive	174 (33.79%)	58 (34.52%)	116 (33.43%)	0.164
Proteinuria (g/d)	1.12 ± 1.19	1.99 ± 1.45	0.16 ± 0.13	<0.001*
Pathological characteristics				
Morphological staging, no. (%)				
Stage I- II	432 (83.88%)	142 (84.52%)	290 (83.57%)	0.157
Stage III- IV	83 (16.12%)	26 (15.48%)	57 (16.43%)	0.138
Crescent formation, no. (%)	44 (8.54%)	15 (8.93%)	29 (8.36%)	0.961
Lesions of FSGS, no. (%)	53 (10.29%)	17 (10.12%)	36 (10.09%)	0.167
Tubular atrophy, no. (%)	289 (56.12%)	92 (54.76%)	197 (56.77%)	0.153
Immunofluorescence				
Immunoglobulin G	489 (94.95%)	160 (95.24%)	329 (94.81%)	0.182
Deposition of C3	391 (75.92%)	133 (77.38%)	258 (74.35%)	0.058
Deposition of C4	93 (18.06%)	32 (19.05%)	61 (17.58%)	0.067
Treatments				
ACEIs or ARBs, no. (%)	421 (81.75%)	139 (82.74%)	282 (81.27%)	0.912
CINs, no. (%)	136 (26.41%)	62 (36.90%)	74 (21.33%)	<0.001*
Remission type				
Complete remission, no. (%)	348 (67.57%)	47 (27.98%)	301 (86.74%)	<0.001*
Partial remission, no. (%)	167 (32.43%)	121 (72.02%)	46 (13.26%)	<0.001*
Follow-up duration (months)	13.68 ± 6.47	8.30 ± 5.33	16.29 ± 5.25	<0.001*

^*^Statistically significant. ^#^Median, Interquartile range. eGFR, Estimated glomerular filtration rate; aPLA2Rab, Anti-phospholipase A2 receptor antibody; FSGS, Focal and segmental glomerulosclerosis; ACEIs, Angiotensin-converting enzyme inhibitors; ARBs, Angiotensin receptor blocker; CNIs, Calcineurin inhibitors; Follow-up duration, The interval from initial remission to the onset of relapse or the last visit, whichever occurred first.

We found widespread use of ACEi or ARB. At least one of these drugs was used in 421/515 patients (81.75%) throughout the clinical course. The treatment regimen used by the majority of patients was glucocorticoids plus cyclophosphamide (278 cases, 53.98%), followed by the use of calcineurin inhibitors (CNIs, with or without any other immunosuppressive drugs, 136 cases, 26.41%), with fewer using rituximab (with or without any other immunosuppressive drugs, 73 cases, 14.17%). Since the CRDS database spans a 24-year period and includes 24 medical centres, and medication regimens vary across generations and centres, we believe that this proportion is not representative of current medication use. 396 (76.89%) patients received more than one immunosuppressive drug, 78 (15.15%) patients received a single immunosuppressive drug, and 41 (7.96%) patients received no immunosuppressive drug. We divided the patients into the CNIs and non-CINs groups. CNIs group referred to the application of CNIs with or without any other immunosuppressive drugs, while Non-CNIs group referred to never administered CNIs. Compared with the non-relapse group, more patients in the relapse group had received CNIs (36.90% vs. 21.33%, *p* < 0.001), as show in Table 1.

### Construction and validation of the prediction model

**Figure 2:**
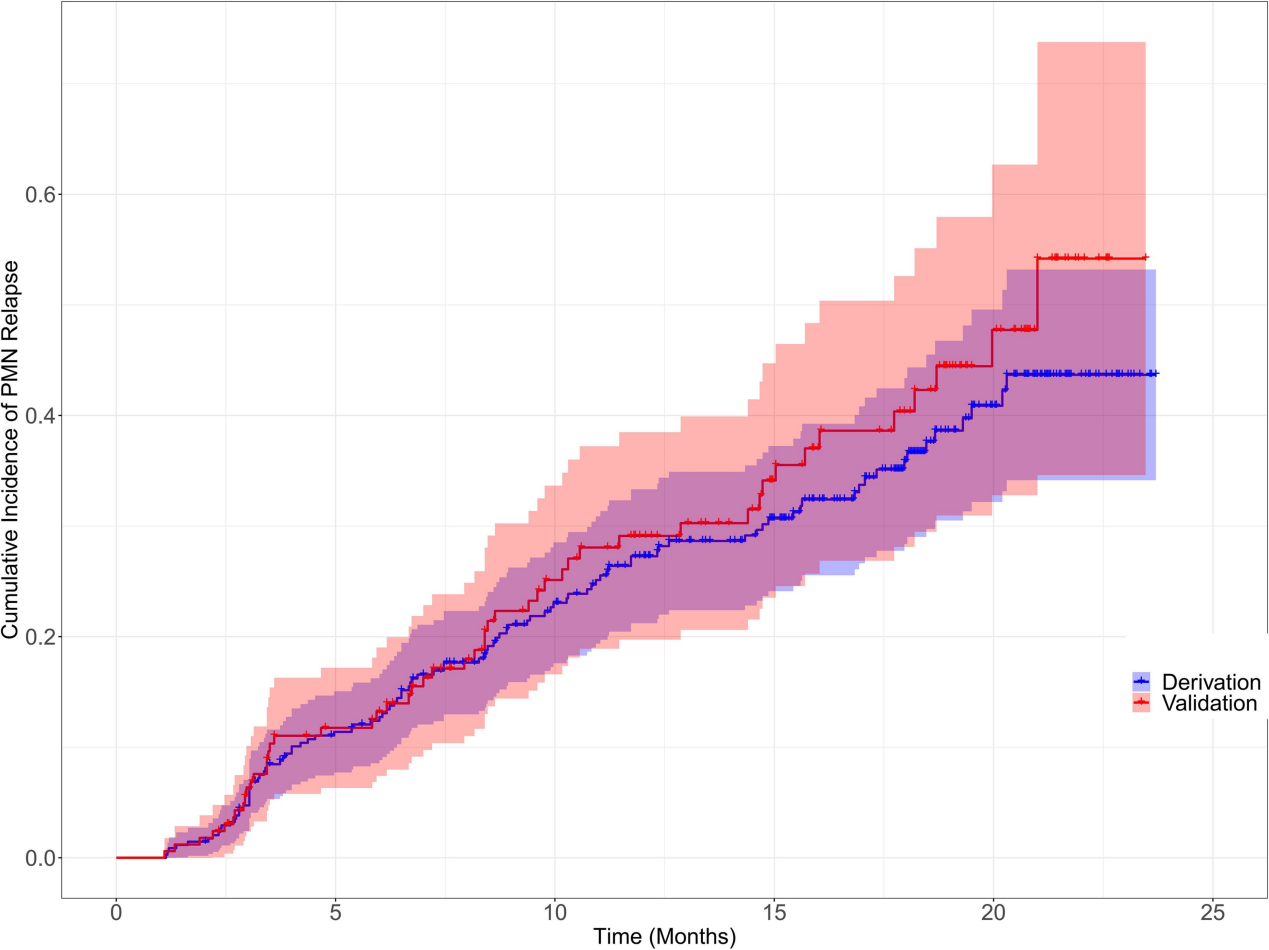
Cumulative incidence of primary membranous nephropathy relapse for the derivation and validation cohorts Cumulative incidence of primary membranous nephropathy relapse for the derivation and validation cohorts were visually compared using the Kaplan–Meier method (Log-rank = 0.85, *p* = 0.357).

Patients were randomly allocated to the derivation (*n* = 361) and validation (*n* = 154) groups in accordance with a ratio of 7:3 using random sampling method, ensuring that the data distribution of the derivation and validation group was consistent. The characteristics of the two groups were presented in Supplementary Table S1. We also visually compared the cumulative relapse incidence curves in derivation and validation cohorts estimated by Kaplan–Meier method, as shown in Figure 2.

Age, proteinuria, Alb, SCr, eGFR, C4, remission type (CR or PR), and treatment regimen (CINs or Non-CINs) were statistically significant between the relapse and non-relapse groups. Correlation analyses were performed on all variables and heatmap were generated (Supplementary Figure S1). Because of the high collinearity between proteinuria and Alb, and between SCR and eGFR, only Alb and eGFR were included in the model. The benefits of these variables were also confirmed using a LASSO Regression (Supplementary Table S2; Supplementary Figure S2). Eventually, these variables and those with clinical significance (aPLAR2ab titers, histological features, and use of ACEi/ARB) were included into Cox proportional hazard models. After multivariate analysis, only Alb (per 1 g/L decrease, hazard ratio [HR] =1.48, 95% confidence interval [CI] 1.29–1.78, *p* < 0.001), eGFR (per 10 mL/min/1.73m^2^ decrease, HR =1.14, 95% CI 0.97–1.49, *p* < 0.001), C4 (per 0.1 g/L increase, HR =1.89, 95% CI 1.32–3.22, *p* = 0.012), PR (HR =2.28, 95%CI 1.74–4.04, *p* < 0.001), and treatment with CINs (HR =1.33, 95%CI 1.04–1.64, *p* < 0.001) were independent risk factors for relapse, as shown in Table 2. There was a trend towards age increasing the risk of relapse, but this was not statistically significant in the multivariate analysis. Our data did not show a statistically significant effect of aPLAR2ab, pathological features, and use of ACEi/ARB on PMN relapse (All *p* > 0.05).

**Table 2:** Risk factors in univariate and multivariate Cox proportional hazard models for PMN relapse.

Variables	Univariate			Multivariate		
	HR	95% CI	*P* value	HR	95% CI	*P* value
Age (per 1 year increase)	1.14	1.08–1.49	0.022^*^	1.01	0.99-1.04	0.117
Partial remission (compared to complete remission)	2.79	1.98–5.23	<0.001^*^	2.28	1.74-4.04	<0.001^*^
Serum albumin (per 1 g/L decrease)	1.74	1.43–2.26	<0.001^*^	1.48	1.29-1.78	<0.001^*^
eGFR (per 10 mL/min/1.73m^2^ decrease)	1.39	1.18–1.82	0.005^*^	1.14	0.97-1.49	0.035^*^
Serum complement 4 (per 0.1 g/L increase)	2.02	1.28–2.79	0.003^*^	1.89	1.32-3.22	0.012^*^
CINs (compared to Non-CNIs)	1.31	1.03–1.51	<0.001^*^	1.33	1.04-1.64	<0.001^*^
aPLAR2ab (per 10RU/mL increase)	1.76	0.69–2.40	0.125	1.32	0.88–1.95	0.522
Stage I- II (compared to stage III- IV)	1.38	0.76–1.63	0.374	1.07	0.94–1.21	0.266
Crescent	1.03	0.65–1.45	0.172	1.04	0.98–1.10	0.165
FSGS	1.82	0.97–3.23	0.112	1.37	1.01–2.13	0.264
Tubular atrophy	1.17	0.56–1.92	0.302	1.03	0.64–1.78	0.886
ACEi/ARB	0.88	0.34–1.45	0.684	0.87	0.46–1.22	0.662

^*^Statistically significant. HR, Hazard ratio; CI, Confidence interval; eGFR, Estimated glomerular filtration rate; CNIs, Calcineurin inhibitors; aPLA2Rab, Anti-phospholipase A2 receptor antibody; FSGS, Focal and segmental glomerulosclerosis; ACEI, Angiotensin-converting enzyme inhibitor; ARB, Angiotensin receptor blocker.

**Figure 3:**
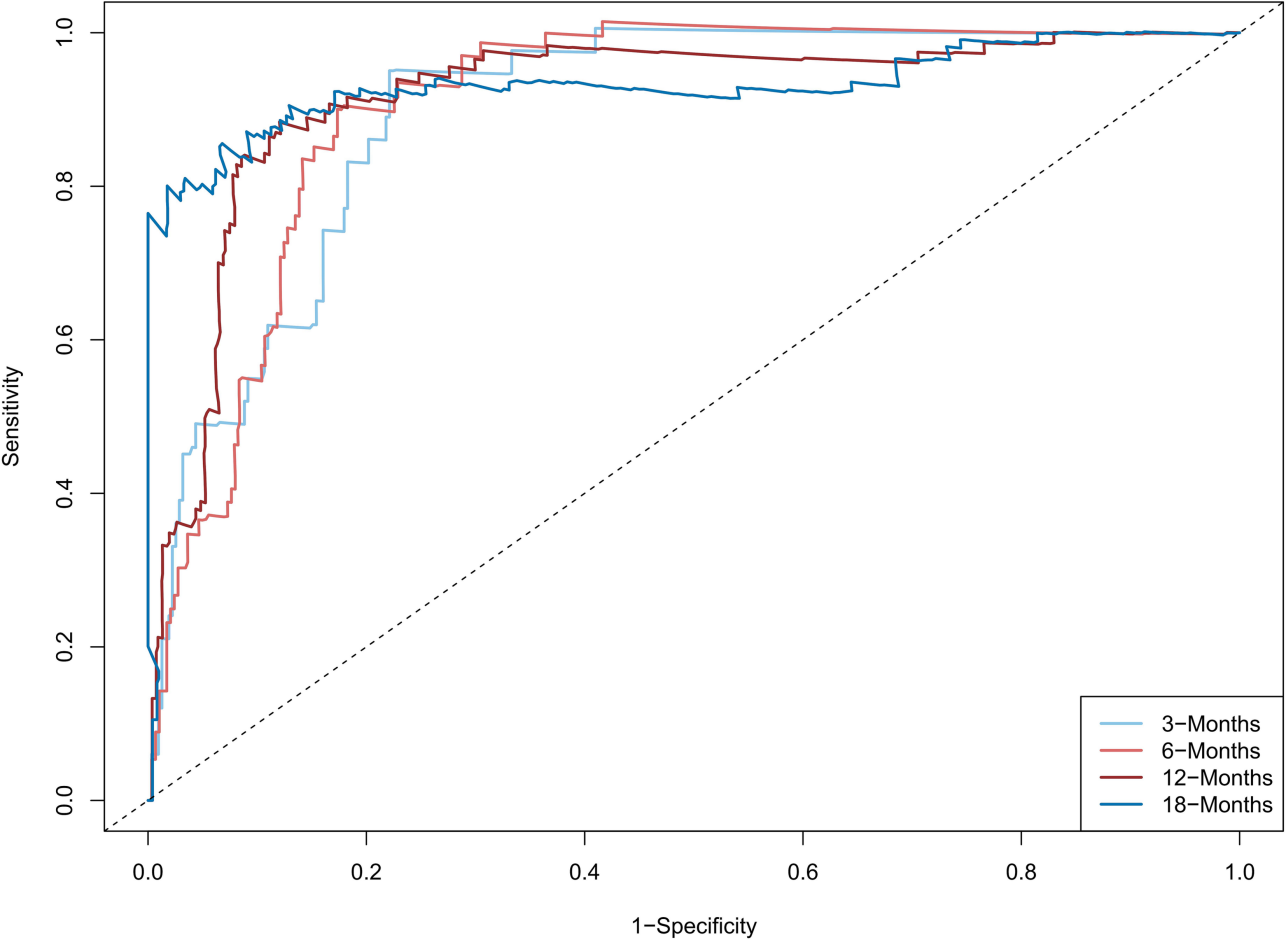
The time-dependent areas under the receiver operating characteristic curve at 3, 6, 12, and 18 months The model combined serum albumin, estimated glomerular filtration rate, serum complement 4, remission type (complete or partial remission), and treatment regimen (calcineurin inhibitors used or not). The time-dependent areas under the receiver operating characteristic curve were 0.898, 0.906, 0.918, and 0.931 at 3, 6, 12, and 18 months, respectively.

**Figure 4:**
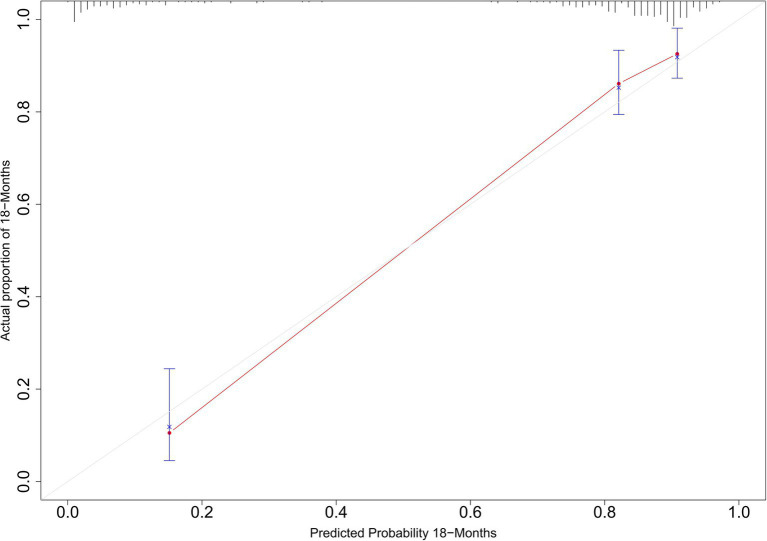
Calibration curves for predicted and actual relapse probabilities in the derivation and validation groups.

The C-statistic were 0.876, 0.875, 0.861, 0.841 and 0.811, 0.805, 0.802, 0.766 in the derivation and validation group at 3, 6, 12, and 18 months, respectively (Supplementary Figure S3). The time-dependent AUROC were 0.898, 0.906, 0.918, and 0.931 at 3, 6, 12, and 18 months, respectively (Figure 3). The aPLA2Rab titers and pathologic features did not substantially improve the model at 3, 6, 12, and 18 months (AUROC 0.901, 0.909, 0.914, and 0.925, respectively) (Supplementary Figure S4). The calibration curves showed that the predicted probabilities and actual probabilities of this model were in good consistency in the derivation and validation groups, as shown in Figure 4. To sum up, these results confirmed that the model had favorable discrimination and calibration predicting the relapse probability of PMN.

### Subgroup and sensitivity analyses

**Figure 5:**
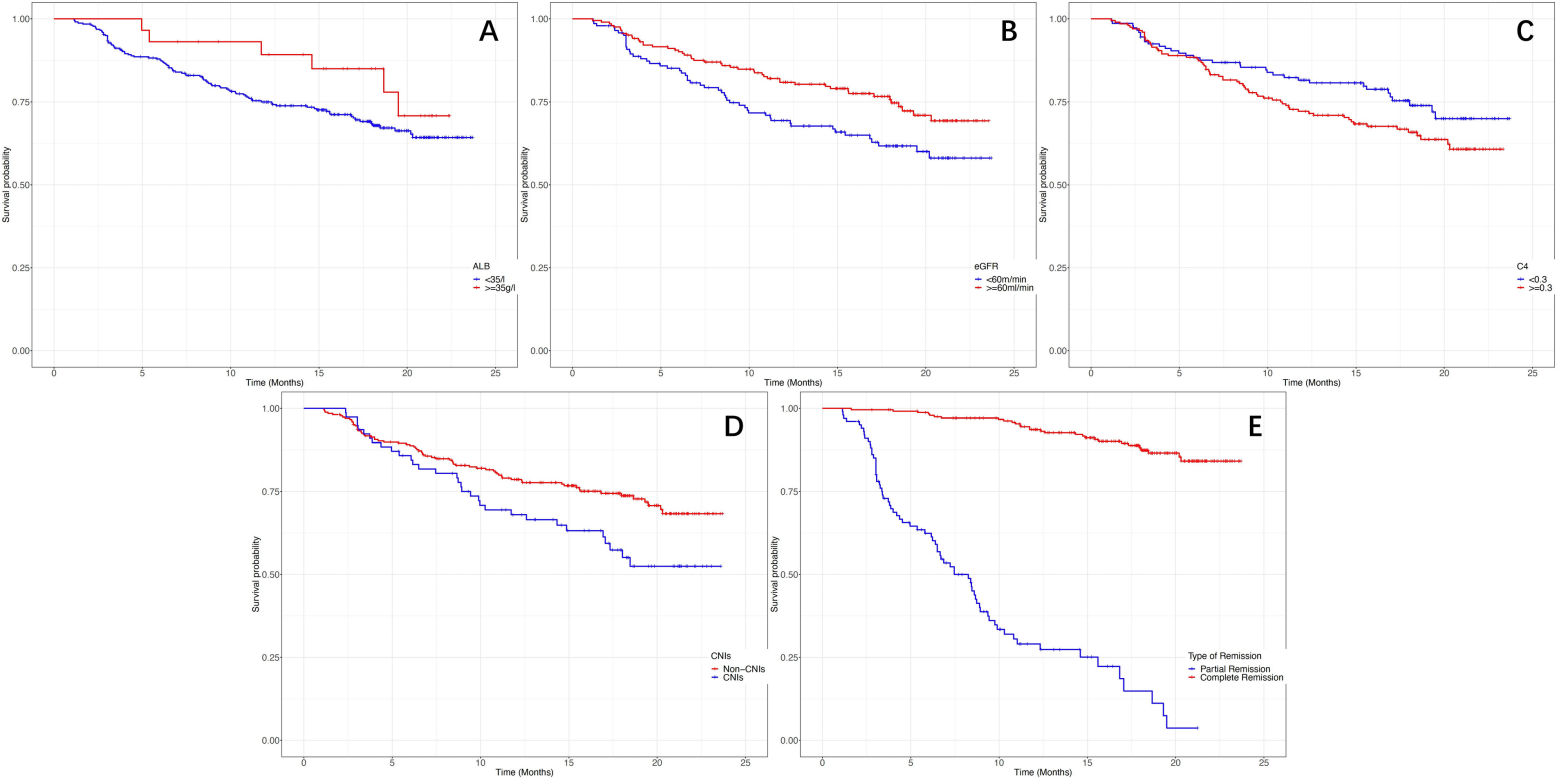
The Kaplan–Meier curves for time to relapse in different subclassifications. (A) Probability of relapse in different serum albumin levels (log-rank χ^2^ = 5.277, *p* = 0.012). (B) Probability of relapse in different estimated glomerular filtration rates (log-rank χ^2^ = 8.506, *p* = 0.008). (C) Probability of relapse in different serum complement 4 levels (log-rank χ^2^ = 6.545, *p* = 0.010). (D) Probability of relapse with or without the use of calcineurin inhibitors (log-rank χ^2^ = 9.545, *p* = 0.003). (E) Probability of relapse in complete or partial remission (log-rank χ^2^ = 12.545, *p* < 0.001).

**Figure 6:**
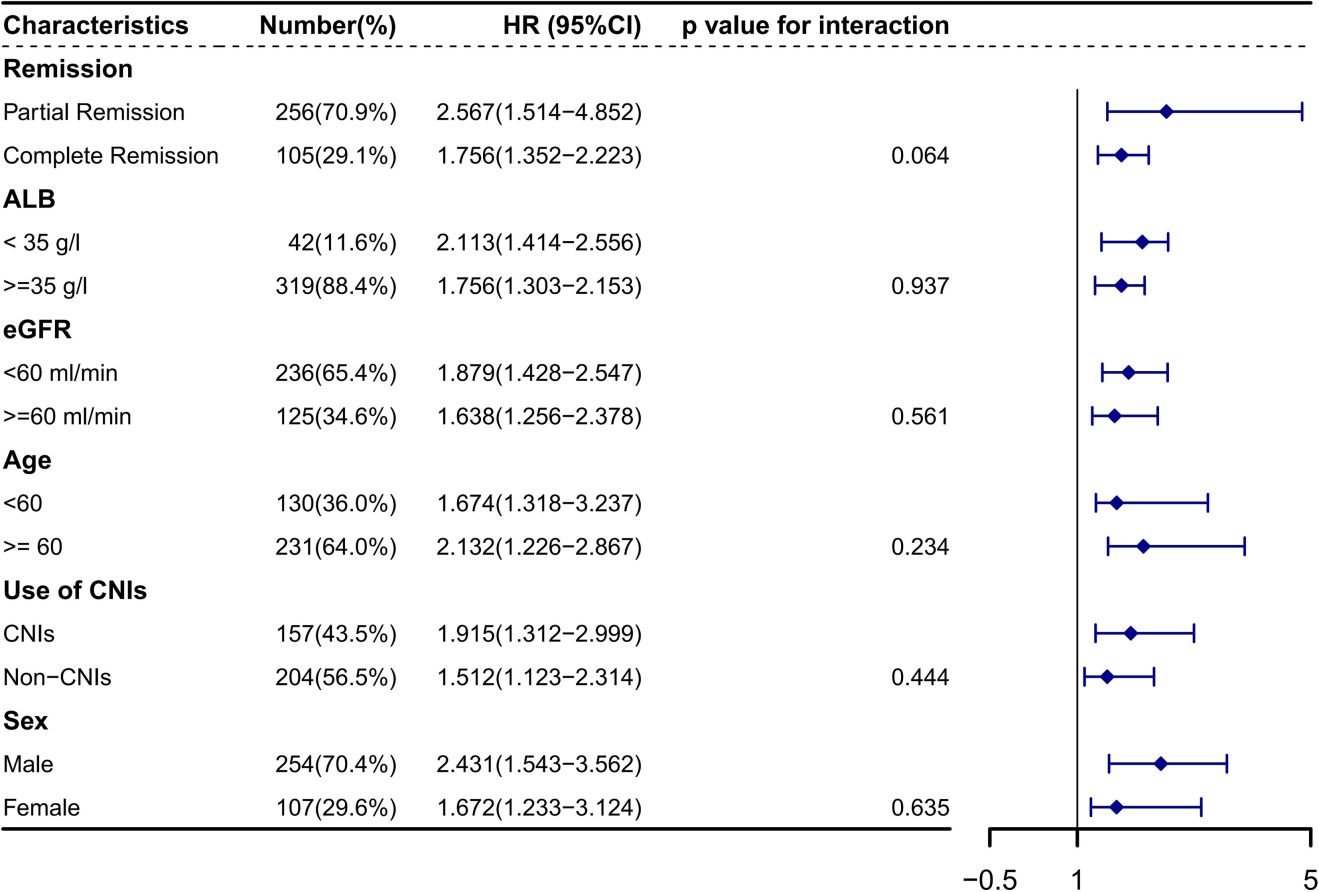
The association between serum complement 4 and primary membranous nephropathy relapse in different subclassifications HR, Hazard ratio; CI, Confidence interval. Alb, Serum albumin; eGFR, Estimated glomerular filtration rate; CNIs, Calcineurin inhibitors. The association between serum complement 4 and relapse could not be modified by age (*p* for interaction = 0.234), sex (*p* = 0.635), Alb (*p* = 0.937), eGFR (*p* = 0.561), remission type (*p* = 0.064), and use of CINs (*p* for interaction = 0.444).

Figure 5 depicts the Kaplan–Meier curves for time to relapse with further subclassifications. The cumulative probability of relapse was higher in the low Alb group (<35 g/L), the low eGFR group (<60 mL/min/1.73m^2^), the high C4 group (≥0.30 g/L), the PR group, and the treatment with CINs group. Additionally, as Alb, eGFR, remission type, and treatment with CINs were common risk factors for relapse, we further analyzed the relationship between C4 and relapse in different subgroups. The results suggested that the association between C4 and relapse could not be modified by age (*p* for interaction = 0.234), sex (*p* = 0.635), Alb (*p* = 0.937), eGFR (*p* = 0.561), remission type (*p* = 0.064), and use of CINs (*p* for interaction = 0.444), as shown in Figure 6.

In sensitivity analyses, restrict the definition of relapse to changes in 24-h urinary protein excretion only, although this leads to a reduction in sample size. Of the initial 515 patients, 237 patients remained for analysis, yet neither the risk factors for relapse nor their HRs changed significantly (Supplementary Table S3).

## Discussion

There remains no proven model for predicting the relapse of PMN. We established this model by combining five common clinical variables (Alb, eGFR, C4, remission type, and treatment regimen). The model showed favorable discrimination and calibration predicting the relapse probability of PMN. Our study may help in improving outcomes in these patients which were managed based on these common clinical variables.

In previous studies, 23–50% of PMN patients relapse after initial remission (7, 9, 15). However, the relapse of PMN after initial remission in China remain unclear. The results of our study showed that 32.62% of patients relapsed in a median of 6.08 months. Our study showed that proteinuria, Alb, eGFR, remission type, C4, and use of CINs at the time of remission were predictors for relapse of PMN. This was expected as proteinuria, Alb and eGFR are well known components of risk assessment for PMN in the KDIGO guidelines (16). Additionally, our results indicate that Alb is a better predictor of relapse than proteinuria, which is supported by the LASSO regression. Our findings were also consistent with the previous studies that achieving CR provides patients with a better probability of being no relapse as compared to patients with PR, in part because PR may not reflect the full disappearance of disease activity (7, 17–19). In our study, histologic characteristics had no impact on the probability of relapse, which was consistent with previous studies (20). Of note, the renal biopsy data were from the onset of PMN, not from the time of remission, as there were no repeat renal biopsies data. Since kidney biopsy provides only a single snapshot of the disease, it may not be able to predict the entire disease course. We believed pathological characteristics at the time of relapse may be more important for predicting the relapse of PMN.

PMN patients with high-normal range serum C4 levels at time of remission were at a greater risk for relapse in our study. Of note, we perform this study by using the de-identified data collected from the CRDS, which consists of regional medical centers across the country with different types of assays and assay providers, but the reference ranges were basically the same, with most of the normal ranges being 0.8–2.0 g/L for C3 and 0.1–0.4 g/L for C4. Complement activation is highly involved in membranous nephropathy, triggering GBM remodeling, podocyte damage and endothelial cell injury (21). Study shows that higher level of C4 was associated with greater risks of renal function progression (22). It is very interesting that C4 is associated elated with relapse or kidney function progression in PMN. Complement can be activated via the classical, lectin and alternative pathways, all of which converge on C3, forming C5b-9 and triggering cellular injury (21, 23). The classical pathway is usually activated by IgG1, IgG2, and IgG3 (24). The lectin pathway can be activated by aberrantly glycosylated IgG4 and induce podocyte injury in a podocyte culture model (25). The fact that early deposits contain more IgG1 and IgG3 whereas later-stage deposits are enriched in IgG4 suggests that the classical complement pathway might initiate disease, which is then propagated by the lectin pathway (24). Moreover, the consistent presence of C4 and mannan-binding lectin in many PMN patients implicates the lectin pathway plays an important role in PMN complement activation and is associated with proteinuria progression and disease activity (26). In another study shown low serum C3 levels was a marker for kidney function progression in PMN patients (27). However, in our study serum C3 levels did not appear to be associated with PMN relapse. The inconsistency of the results may be due to their small sample size (99 patients), single centre, long follow-up time (6.2 years) and different endpoints. The consumption of complement was mentioned in several studies as a marker of disease activity, such as in thrombotic microangiopathies and lupus (28–30). But the complement activation pathway in PMN may be different from that way. The study showed that low serum C3 levels and high serum C4 levels were associated with a higher risk of poor renal prognosis in patients with microscopic polyangiitis (31). There may be production and consumption balance differences between C3 and C4, with increased C4 production and decreased C4 consumption in PMN, resulting in high serum C4 levels that may be associated with poor renal prognosis. Activation of the complement system is a key contributor to the development of PMN, further molecular understanding of the underlying pathological mechanisms is needed. While there is promising preliminary experience with the use of complements and their activation products in disease monitoring and prognostication, these approaches will have to be validated in well-defined patient cohorts (32, 33).

The use of ACEi/ARB varied (39–95%) in previous studies (34, 35). Our study reveals that in clinical practice, Chinese clinicians tend to use ACEi/ARB widely (81.75%) for supportive therapy. However, our data failed to show a significant effect of ACEi/ARB on relapse prevention, which may be masked by the high prevalence of their use. In China, first-line immunosuppressive therapy included a cyclic regimen of alternating glucocorticoids and cyclophosphamide, the second most used treatment was rituximab-based therapy, and the last choice was CNIs-based therapy. CNIs have immunosuppressive effects, directly acting on kidney podocytes and indirectly affecting B-cell function (36, 37), thereby reducing proteinuria and inducing a higher remission rate. However, similar to previous studies, our study showed treatment with CINs was also associated with relapse (38–40). Therefore, CNIs are considered an alternative to cytotoxic-based or rituximab-based regimens. On the other hand, clinicals usually choose to use CNIs only when the cytotoxic-based or rituximab-based regimens show poor efficacy, so it could also be that the patients *per se* are sicker to be more prone to relapse, rather than the CNIs causing the relapse.

High titers of aPLA2Rab have been associated with reduced likelihood of spontaneous remission, poor treatment response, and poor clinical outcome (41). In our study, although the median aPLA2Rab titer at the initiation of remission was higher in relapse group, the difference did not reach significance. Moreover, The time-dependent AUROC showed that aPLA2Rab did not substantially improve the prediction model. It may be that not all patients in our cohort were aPLA2Rab-associated MN. Additionally, the titers of aPLA2Rab are dynamic (42), as patients are at different phases of the disease, some entering immunological remission while others having increasing immunological activity, one cannot rely on snapshot measurement of aPLA2Rab titers to predict the relapse, but rather on the trajectory of aPLA2Rab titers (43). Future studies are needed to determine the possible role of longitudinal monitoring of aPLA2Rab as a prognostic biomarker of relapse.

Our study is a large multicenter, regionwide and nationwide study. It is the first study to investigate the association between clinical features at the time of remission and relapse in PMN. In addition, our study included anti-PLA2R antibody titers and histopathological features, which were commonly lacking in previous studies. Our study has its limitations. (1) As an observational retrospective study, we could not avoid its inherent limitations, there may be some residual confounding that may affect the results. (2) Patients in CRDS are predominantly Chinese, the generalizability of our results may be limited. (3) All clinical variables were not dynamically observed during the follow-up period. (4) We did not perform subgroup analyses of patients in spontaneous remission. Although we tried our best to record all medications during the course of the disease, because the CRDS database spans 24 years, there may still be medications that were not recorded. In China, patients want their doctors to treat them as early as possible, not just with conservative supportive care, and then wait for ‘spontaneous remission’. Therefore, the number of patients in ‘spontaneous remission’ was small and we could not analyze this subgroup further. Improved the relapse prediction model will require a more complex model that would include repeated measurements of clinical variables.

## Conclusion

Our study confirms the well-known low Alb and eGFR, PR, and treatment of CNIs at the time of remission as risk factors for PMN relapse. In addition, it is the first study to show serum C4 is associated with PMN relapse. We suggest that complement-targeted therapies may be a potential therapy to prevent PMN relapse. Large-scale multicenter prospective studies may have the potential to verify our findings and the mechanism of this clinical phenomenon remains to be elucidated.

## Data Availability

The raw data supporting the conclusions of this article will be made available by the authors, without undue reservation.

## Footnotes

^1^ http://www.crds-network.org.cn/#/

## Supplementary material

SUPPLEMENTARY FIGURE S1 Correlation of all potential risk variables in the heatmap. CNIs, Calcineurin inhibitors; ACEI, Angiotensin-converting enzyme inhibitor; ARB, Angiotensin receptor blocker; FSGS, Focal and segmental glomerulosclerosis; SBP, Systolic blood pressure; DPB, Diastolic blood pressure; BMI, Body mass index; eGFR, Estimated glomerular filtration rate.

SUPPLEMENTARY FIGURE S2 LASSO regression for all potential risk variables.

SUPPLEMENTARY FIGURE S3 The C-statistic in the derivation and validation group at 3, 6, 12, and 18 months. The C-statistic were 0.876, 0.875, 0.861, 0.841 and 0.811, 0.805, 0.802, 0.766 in the derivation and validation group at 3, 6, 12, and 18 months, respectively.

SUPPLEMENTARY FIGURE S4 The time-dependent areas under the receiver operating characteristic curve at 3, 6, 12, and 18 months (the model plus aPLA2Rab and pathologic features). The model combined serum albumin, estimated glomerular filtration rate, serum complement 4, remission type (complete or partial remission), treatment regimen (calcineurin inhibitors used or not), aPLA2Rab titers, morphological staging, crescent, focal and segmental glomerulosclerosis, and tubular atrophy. The time-dependent areas under the receiver operating characteristic curve were 0.901, 0.909, 0.914, and 0.925, respectively.
