# Supplementary material for: The association between serum complement 4 and relapse of primary membranous nephropathy: a multicenter retrospective cohort study
Source: Front Med (Lausanne). 2024 Nov 11;11:1451677. doi: 10.3389/fmed.2024.1451677 (PMC11586214; doi:10.3389/fmed.2024.1451677)
Supplement: Supplementary file 1 [file Table_1.DOCX]

| Supplementary Table 1 The characteristics of the derivation and validation groups | | | | |
| --- | --- | --- | --- | --- |
|  | All patients | Derivation group | Validation group | P value |
|  | (n=515) | (n=361) | (n=154) |  |
| Demographic characteristics |  |  |  |  |
| Male no. (%) | 354 (68.74%) | 246 (68.14%) | 108 (70.13%) | 0.313 |
| Age (years) | 48.21 ± 13.57 | 47.75 ± 13.92 | 49.17 ±12.82 | 0.268 |
| BMI (kg/m^2^) | 25.11 ± 3.11 | 25.12 ± 3.38 | 25.11 ± 3.11 | 0.977 |
| Comorbid­ities |  |  |  |  |
| Hypertension no. (%) | 198 (38.45%) | 140 (38.78%) | 58 (37.66%) | 0.310 |
| Diabetes mellitus no. (%) | 76 (14.76%) | 50 (13.86%) | 26 (16.88%) | 0.326 |
| SBP (mmHg) | 131.12 ± 16.97 | 130.69 ± 17.46 | 132.0 ± 15.91 | 0.414 |
| DBP (mmHg) | 80.95 ± 10.71 | 80.45 ± 11.06 | 82.00 ± 9.90 | 0.124 |
| Laboratory tests |  |  |  |  |
| Hemoglobin (g/L) | 131.95 ± 21.61 | 131.31 ± 21.49 | 133.28 ± 21.88 | 0.333 |
| Serum albumin (g/L) | 22.43 ± 5.73 | 21.75 ± 5.80 | 23.84 ± 5.32 | 0.229 |
| Serum creatinine (μmol/L) | 85.99 ± 42.68 | 85.61 ± 40.14 | 86.78 ± 47.62 | 0.783 |
| eGFR (mL/min/1.73 m^2^) | 91.00 ± 26.06 | 91.41 ± 26.47 | 90.14 ± 25.23 | 0.604 |
| Triglyceride (mmol/L) | 3.34 ± 8.82 | 3.11 ±2.32 | 3.61 ± 7.35 | 0.268 |
| Total cholesterol (mmol/L) | 8.58 ± 2.97 | 8.84 ± 2.95 | 8.11 ±2.77 | 0.375 |
| Serum complement 3 (g/L) | 1.13 ± 0.31 | 1.14 ± 0.39 | 1.11 ± 0.24 | 0.308 |
| Serum complement 4 (g/L) | 0.29 ± 0.11 | 0.29 ± 0.11 | 0.28 ± 0.09 | 0.118 |
| aPLA2Rab titer (RU/ml) ^#^ | 25.25 (18.83, 49.87) | 25.12 (18.02, 50.2) | 26.14 (17.57, 48.63) | 0.358 |
| Proteinuria (g/d) | 1.12 ± 1.19 | 1.19 ± 1.32 | 1.04 ± 1.22 | 0.636 |
| Pathological characteristics |  |  |  |  |
| Morphological staging, no. (%) |  |  |  |  |
| stage I- II | 432 (83.88%) | 302 (83.66%) | 130 (84.42%) | 0.832 |
| stage III- IV | 83 (16.12%) | 61 (16.89%) | 22 (14.29%) | 0.322 |
| Crescent formation, no. (%) | 44 (8.54%) | 31 (8.59%) | 13 (8.44%) | 0.683 |
| Lesions of FSGS, no. (%) | 53 (10.29%) | 17 (10.80%) | 14 (9.09%) | 0.696 |
| Tubular atrophy, no. (%) | 289 (56.12%) | 199 (55.12%) | 90 (58.44%) | 0.733 |
| Treatments |  |  |  |  |
| ACEIs or ARBs, no. (%) | 421 (81.75%) | 291 (80.61%) | 130 (84.42%) | 0.247 |
| CINs, no. (%) | 136 (26.41%) | 94 (26.04%) | 42 (27.27%) | 0.302 |
| Remission type |  |  |  |  |
| Complete remission, no. (%) | 348 (67.57%) | 249 (68.98%) | 99 (64.29%) | 0.832 |
| Partial remission, no. (%) | 167 (32.43%) | 112 (31.02%) | 55 (35.71%) | 0.895 |
| Follow-up duration (months) | 13.68 ± 6.47 | 13.31 ± 7.87 | 14.09 ± 6.53 | 0.141 |
| ^#^, Median, Interquartile range. BMI, Body mass index; SBP, Systolic blood pressure; DPB, Diastolic blood pressure; eGFR, Estimated glomerular filtration rate; aPLA2Rab, Anti-phospholipase A2 receptor antibody; FSGS, Focal and segmental glomerulosclerosis; ACEIs, Angiotensin-converting enzyme inhibitors; ARBs, Angiotensin receptor blocker; CNIs, Calcineurin inhibitors; Follow-up duration, The interval from initial remission to the onset of relapse or the last visit, whichever occurred first. | | | | |

| Supplementary Table 2 The results of LASSO regression for potential risk variables | |
| --- | --- |
| Variables | Index |
| Complete remission | 0.57390307 |
| Serum albumin | 0.00477451 |
| eGFR | 0.00250344 |
| Age | -0.0413313 |
| Proteinuria | -0.063383 |
| Serum complement 4 | -0.049285 |
| Serum creatinine | -0.2322085 |
| CNIs | -0.53306 |
| aPLA2Rab | 0 |
| Serum complement 3 | 0 |
| Male | 0 |
| Hypertension | 0 |
| Diabetes mellitus | 0 |
| SBP | 0 |
| DBP | 0 |
| Hemoglobin | 0 |
| Triglyceride | 0 |
| Total cholesterol | 0 |
| Stage I- II | 0 |
| Crescent | 0 |
| FSGS | 0 |
| Tubular atrophy | 0 |
| eGFR, Estimated glomerular filtration rate; CNIs, Calcineurin inhibitors; aPLA2Rab, Anti-phospholipase A2 receptor antibody; SBP, Systolic blood pressure; DPB, Diastolic blood pressure; FSGS, Focal and segmental glomerulosclerosis. | |

| Supplementary Table 3 Sensitivity analysis of relapse definition restricted to changes in 24-hour urinary protein | | | |
| --- | --- | --- | --- |
| Variables | Multivariate model | | |
|  | HR | 95% CI | P value |
| Age (per 1 year increase) | 1.09 | 0.87-1.13 | 0.347 |
| Partial remission (compared to complete remission) | 2.68 | 1.31-4.56 | <0.001^*^ |
| Serum albumin (per 1g/L decrease) | 1.76 | 1.09-2.13 | <0.001^*^ |
| eGFR (per 10ml/min/1.73m^2^ decrease) | 1.32 | 1.01-1.97 | 0.004^*^ |
| Serum complement 4 (per 0.1g/L increase) | 1.68 | 1.30-2.98 | 0.003^*^ |
| CINs (compared to Non-CNIs) | 1.78 | 0.87-2.89 | 0.012^*^ |
| aPLAR2ab (per 10RU/ml increase) | 1.43 | 0.56-2.45 | 0.879 |
| Stage I- II (compared to stage III- IV) | 1.00 | 0.90-1.52 | 0.387 |
| Crescent | 0.97 | 0.67-1.23 | 0.962 |
| FSGS | 1.45 | 1.00-2.56 | 0.142 |
| Tubular atrophy | 1.31 | 0.96-2.70 | 0.526 |
| ACEi/ARB | 0.99 | 0.87-1.41 | 0.262 |
| *, Statistically significant. HR, Hazard ratio; CI, Confidence interval; eGFR, Estimated glomerular filtration rate; CNIs, Calcineurin inhibitors; aPLA2Rab, Anti-phospholipase A2 receptor antibody; FSGS, Focal and segmental glomerulosclerosis; ACEI, Angiotensin-converting enzyme inhibitor; ARB, Angiotensin receptor blocker. | | | |

| Supplementary Tab 4 The baseline characteristics at the time of PMN diagnosis | | | |
| --- | --- | --- | --- |
|  | Relapse group | Non-relapse group | P value |
|  | (n=168) | (n=347) |  |
| Demographic characteristics |  |  |  |
| Male no. (%) | 117 (69.64%) | 237 (68.30%) | 0.387 |
| Age (years) | 51.47 ± 12.37 | 46.63 ± 13.86 | <0.001* |
| BMI (kg/m^2^) | 25.38 ± 3.20 | 24.98 ± 3.06 | 0.178 |
| Comorbid­ities |  |  |  |
| Hypertension no. (%) | 67 (39.88%) | 131 (37.75%) | 0.310 |
| Diabetes mellitus no. (%) | 30 (17.86%) | 46 (13.26%) | 0.212 |
| SBP (mmHg) | 133.56 ± 18.31 | 130.79 ± 16.22 | 0.086 |
| DBP (mmHg) | 81.37 ± 11.22 | 80.61 ± 10.55 | 0.296 |
| Laboratory tests |  |  |  |
| Hemoglobin (g/L) | 130.32 ± 21.21 | 132.51 ± 21.90 | 0.762 |
| Serum albumin (g/L) | 22.21 ± 5.40 | 22.53 ± 5.89 | 0.546 |
| Serum creatinine (μmol/L) | 90.74 ± 39.9 | 83.76 ± 43.8 | 0.069 |
| eGFR (mL/min/1.73 m^2^) | 85.67 ± 25.6 | 93.64 ± 25.9 | 0.001* |
| Triglyceride (mmol/L) | 3.11 ± 2.12 | 3.45 ± 1.97 | 0.566 |
| Total cholesterol (mmol/L) | 8.65 ± 2.80 | 8.58 ± 2.97 | 0.773 |
| Serum complement 3 (g/L) | 1.12 ± 0.42 | 1.13 ± 0.31 | 0.935 |
| Serum complement 4 (g/L) | 0.28 ± 0.09 | 0.29 ± 0.11 | 0.032* |
| aPLA2Rab titer (RU/ml) ^#^ | 137.74 (87.96, 180.79) | 130.10 (79.71, 173.59) | 0.129 |
| aPLA2Rab positive | 140 (83.31%) | 258 (82.13%) | 0.164 |
| Proteinuria (g/d) | 5.99 ± 1.45 | 4.46 ± 1.63 | <0.001* |
| Pathological characteristics |  |  |  |
| Morphological staging, no. (%) |  |  |  |
| stage I- II | 432 (83.88%) | 142 (84.52%) | 290 (83.57%) |
| stage III- IV | 83 (16.12%) | 26 (15.48%) | 57 (16.43%) |
| Crescent formation, no. (%) | 44 (8.54%) | 15 (8.93%) | 29 (8.36%) |
| Lesions of FSGS, no. (%) | 53 (10.29%) | 17 (10.12%) | 36 (10.09%) |
| Tubular atrophy, no. (%) | 289 (56.12%) | 92 (54.76%) | 197 (56.77%) |
| Immunofluorescence |  |  |  |
| Immunoglobulin G | 489 (94.95%) | 160 (95.24%) | 329 (94.81%) |
| Deposition of C3 | 391 (75.92%) | 133 (77.38%) | 258 (74.35%) |
| Deposition of C4 | 93 (18.06%) | 32 (19.05%) | 61 (17.58%) |
| Treatments |  |  |  |
| ACEIs or ARBs, no. (%) | 139 (82.74%) | 282 (81.27%) | 0.912 |
| CINs, no. (%) | 62 (36.90%) | 74 (21.33%) | <0.001* |
| ^*^, Statistically significant. ^#^, Median, Interquartile range. BMI, Body mass index; SBP, Systolic blood pressure; DPB, Diastolic blood pressure; eGFR, Estimated glomerular filtration rate; aPLA2Rab, Anti-phospholipase A2 receptor antibody; FSGS, Focal and segmental glomerulosclerosis; ACEIs, Angiotensin-converting enzyme inhibitors; ARBs, Angiotensin receptor blocker; CNIs, Calcineurin inhibitors; Follow-up duration, The interval from initial remission to the onset of relapse or the last visit, whichever occurred first. | | | |
