# Supplementary figures and images for: The association between serum complement 4 and relapse of primary membranous nephropathy: a multicenter retrospective cohort study
Source: Front Med (Lausanne). 2024 Nov 11;11:1451677. doi: 10.3389/fmed.2024.1451677 (PMC11586214; doi:10.3389/fmed.2024.1451677)

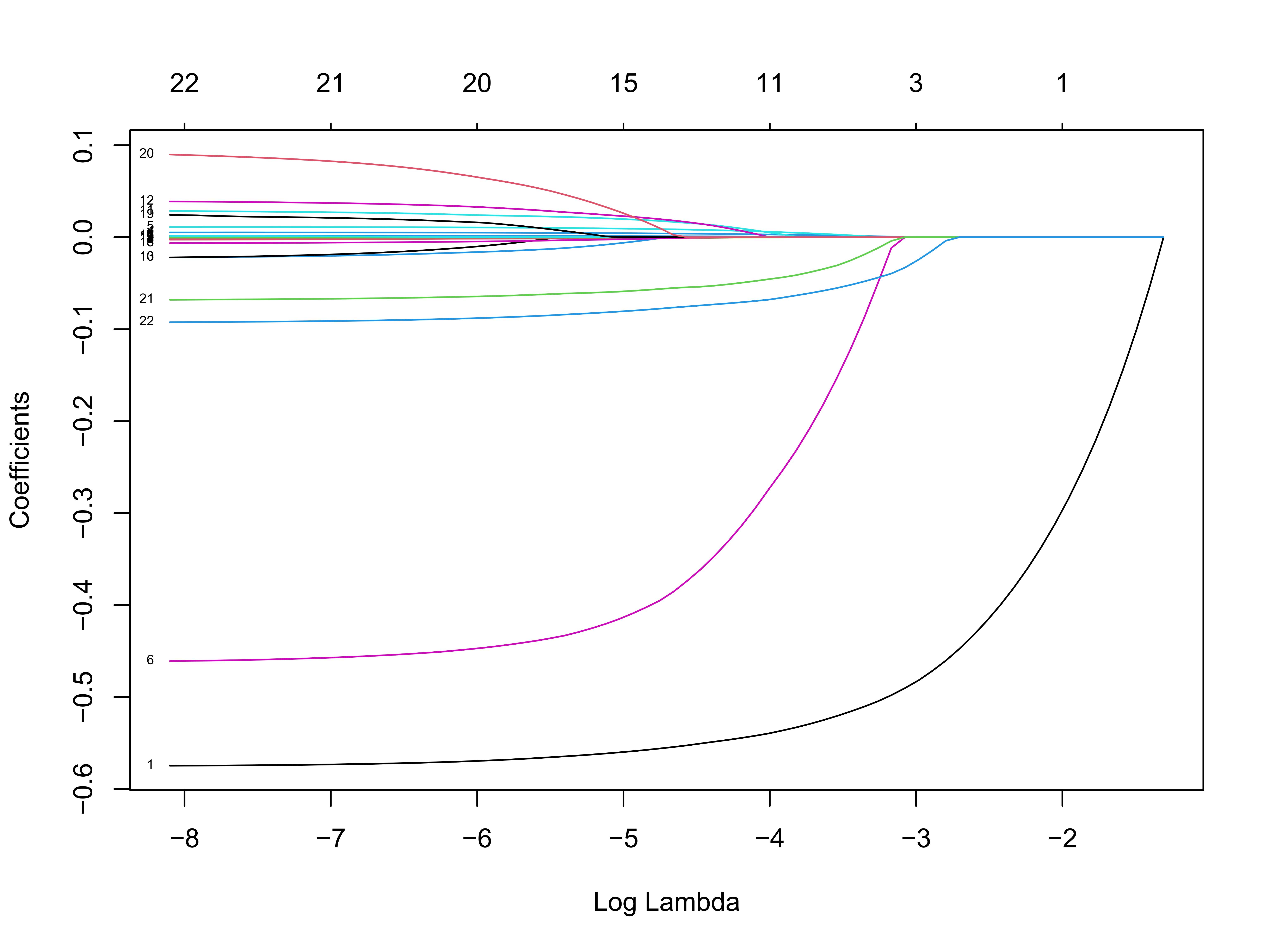

Supplement: SUPPLEMENTARY FIGURE S2 — LASSO regression for all potential risk variables. [file Image_2.JPEG]

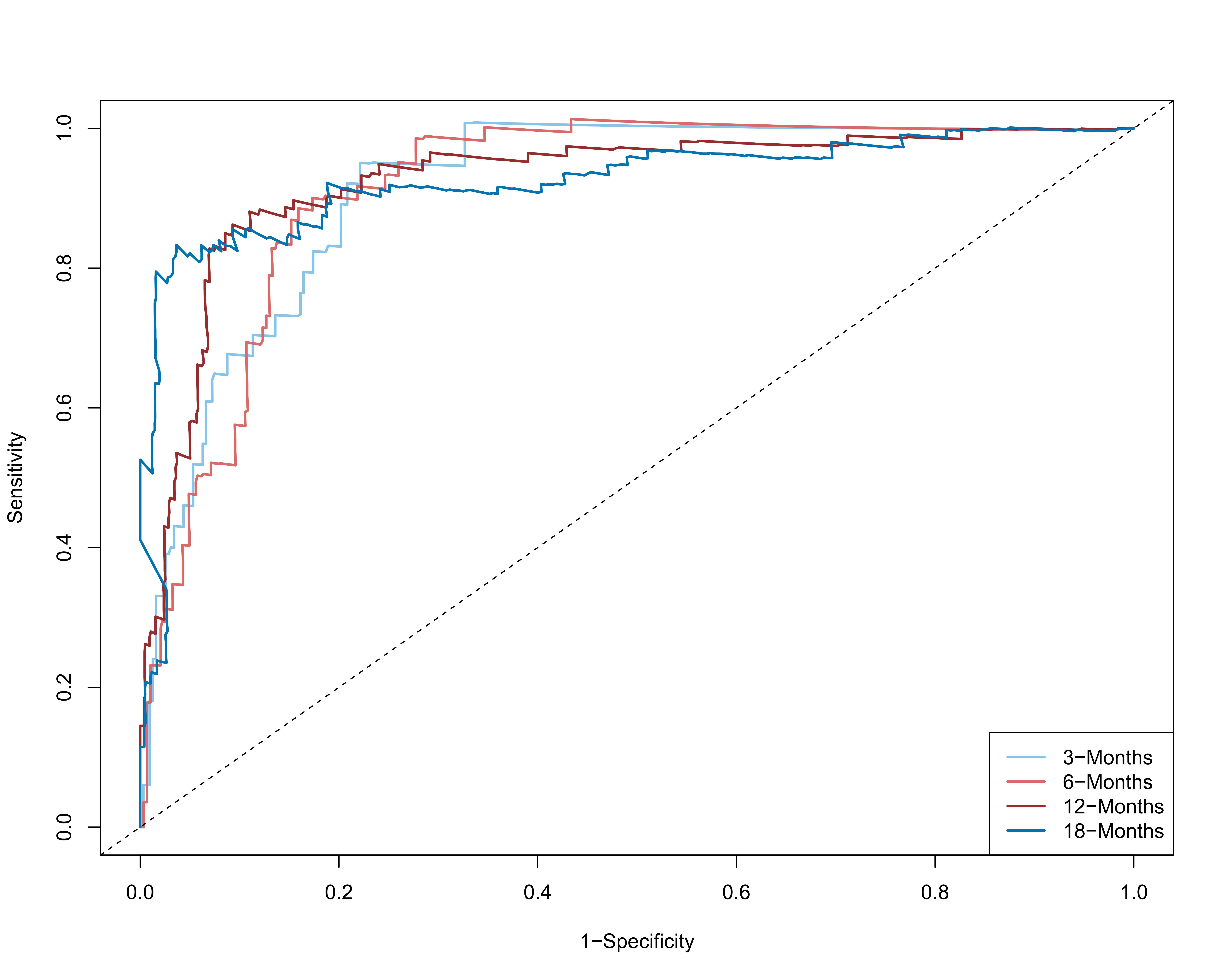

Supplement: SUPPLEMENTARY FIGURE S4 — The time-dependent areas under the receiver operating characteristic curve at 3, 6, 12, and 18 months (the model plus aPLA2Rab and pathologic features). The model combined serum albumin, estimated glomerular filtration rate, serum complement 4, remission type (complete or partial remission), treatment regimen (calcineurin inhibitors used or not), aPLA2Rab titers, morphological staging, crescent, focal and segmental glomerulosclerosis, and tubular atrophy. The time-dependent areas under the receiver operating characteristic curve were 0.901, 0.909, 0.914, and 0.925, respectively. [file Image_4.JPEG]
